# Prevalence and resistance spectrum of *ahpC* mutations in isoniazid-resistant *Mycobacterium tuberculosis* isolates

**DOI:** 10.1128/spectrum.02496-25

**Published:** 2026-03-19

**Authors:** Xiaomeng Wu, Huiwen Zheng, Yiyi Chen, Bing Zhao, Feina Li, Hui Xia, Jing Xiao, Hui Qi, Weiwei Jiao, Kaixia Mi, Xichao Ou, Yanlin Zhao, Lin Sun

**Affiliations:** 1 Laboratory of Respiratory Diseases, Beijing Pediatric Research Institute, Beijing Children’s Hospital, Capital Medical University, Beijing Key Laboratory of Core Technologies for the Prevention and Treatment of Emerging Infectious Diseases in Children, Key Laboratory of Major Diseases in Children, Ministry of Education, National Clinical Research Center for Respiratory Diseases, National Center for Children’s Health 38038 , Beijing, China; 2 National Key Laboratory of Intelligent Tracking and Forecasting for Infectious Diseases, National Center for Tuberculosis Control and Prevention, Chinese Centre for Disease Control and Prevention 34720 , Beijing, China; 3 Laboratory of Pathogen Microbiology and Immunology, Institute of Microbiology, Chinese Academy of Sciences, Medical School, University of Chinese Academy of Sciences 74519, Beijing, China; Assistance Publique-Hopitaux de Paris Universite Paris Saclay, Clamart, France

**Keywords:** *Mycobacterium tuberculosis*, drug resistance, isoniazid, AhpC, gene mutation

## Abstract

To characterize the mutation profile of the *ahpC* gene in isoniazid resistance (INH^r^) *Mycobacterium tuberculosis* (MTB) isolates and to evaluate the correlation between specific mutations and resistance levels, a total of 1,337 INH^r^ MTB isolates were collected through the Chinese Drug Resistance Surveillance Program (2013–2020). The minimum inhibitory concentrations (MICs) for INH, rifampicin (RIF), and ethionamide were determined by broth microdilution, followed by whole-genome sequencing analysis. Among 1,337 INH^r^*-*MTB isolates, 45.5% (608/1,337) were multidrug-resistant (MDR), and 54.5% (729/1,337) were RIF-sensitive cases, with lineage 2 predominating (1,013/1,337, 75.8%). Resistance mutations were identified in 79.1% (1,058/1,337) of strains, primarily *katG* Ser315Thr (65.5%, 695/1,058) and *inhA* C-777T (19.7%, 208/1,058). Notably, 5.3% (56/1,058) isolates harbored standalone *ahpC* mutations, with 83.3% (5/6) C-81T, 60.0% (9/15) G-48A, 57.1% (4/7) C-54T, 50.0% (3/6) C-57T, and 42.1% (8/19) C-52T mutations showing high-level INH resistance. Accordingly, 91.7% (11/12) dual *katG*^315^+*inhA* mutations conferred high-level INH resistance, while *ahpC* C-57T mutants universally exhibited MDR. The *ahpC* mutations are associated with high-level INH resistance in variants without concurrent *katG* or *inhA* mutations. This finding significantly advances our understanding of tuberculosis resistance profiling, enabling more comprehensive detection of INH^r^-MTB and optimizing therapeutic strategies.

**IMPORTANCE:**

Among INH-resistant MTB clinical isolates, mutations in the *ahpC* promoter region have been considered to occur in combination with other mutations, such as *katG* and *inhA*, in a compensatory role. While the *ahpC* mutations have been incorporated into the World Health Organization (WHO)-recommended rapid diagnostic test, Xpert MTB/extensively drug-resistant tuberculosis (XDR), it has been still ambiguous about the standalone effects on INH resistance spectrum. Our findings demonstrate that the *ahpC* mutations are associated with high-level INH resistance in variants without concurrent *katG* or *inhA* mutations. This finding significantly advances our understanding of TB resistance profiling, enabling a more comprehensive detection of INH^r^-MTB and optimizing therapeutic strategies.

## INTRODUCTION

Tuberculosis (TB) continues to pose a significant global health challenge, with the emergence of drug-resistant TB (DR-TB) presenting major obstacles to effective disease control. According to the World Health Organization (WHO), an estimated 10.8 million new TB cases were reported worldwide, including approximately 1.25 million deaths and 400,000 (3.7%) new cases of multidrug-resistant (MDR)/rifampicin-resistant (RR) TB, which has remained stable since 2020 (1). Notably, China accounts for 7.3% of global MDR/RR-TB cases, ranking third worldwide (1). Given its high mortality rate, complex treatment requirements, and potential for sustained transmission, MDR-TB has been designated by WHO as a critical priority among antibiotic-resistant bacterial pathogens (2). Of particular concern is the emergence of isoniazid resistance (INH^r^), which not only represents a critical initial step toward MDR-TB development but also directly impacts clinical outcomes (3). It is evidenced by alarmingly high treatment failure rates (18–44%) observed in patients with INH-mono-resistant TB, significantly complicating disease management (3–5). Additionally, the presence of cross-resistance between ethionamide (ETO) and INH underscores the urgent need for rapid and accurate detection of INH resistance mutations to guide therapeutic decision-making and prevention strategies.

INH resistance in *Mycobacterium tuberculosis* (MTB) primarily arises from mutations in the *katG* gene and *inhA* gene or its promoter region (6). Although certain drug-resistant mutations, such as those in the *ahpC* promoter region, typically occur in combination with other mutations and are rarely found as standalone variants, they have been incorporated into the WHO-recommended rapid diagnostic test, Xpert MTB/ extensively drug-resistant tuberculosis (XDR) (7–11). The *ahpC* gene encodes alkyl hydroperoxide reductase (AhpC), an enzyme that detoxifies organic peroxides and protects bacterial cells from oxidative damage caused by KatG inactivation (7–11). However, the precise role of *ahpC* mutations in INH resistance remains controversial—whether they merely compensate for functional defects in KatG mutants or directly confer INH resistance requires further investigation (12).

As a reliable, rapid, and increasingly cost-effective technology, whole-genome sequencing (WGS) can predict drug resistance spectrum of MTB, particularly for first-line anti-TB drugs, allowing timely initiation of appropriate treatment regimens (13). The current study aims to characterize the mutation profile of the *ahpC* gene in INH^r^-MTB isolates using WGS and evaluate the correlation between specific mutations and resistance levels, which will contribute to improving diagnostic accuracy and treatment outcomes for INH-resistant TB patients.

## MATERIALS AND METHODS

### Isolate selection

**Fig 1:**
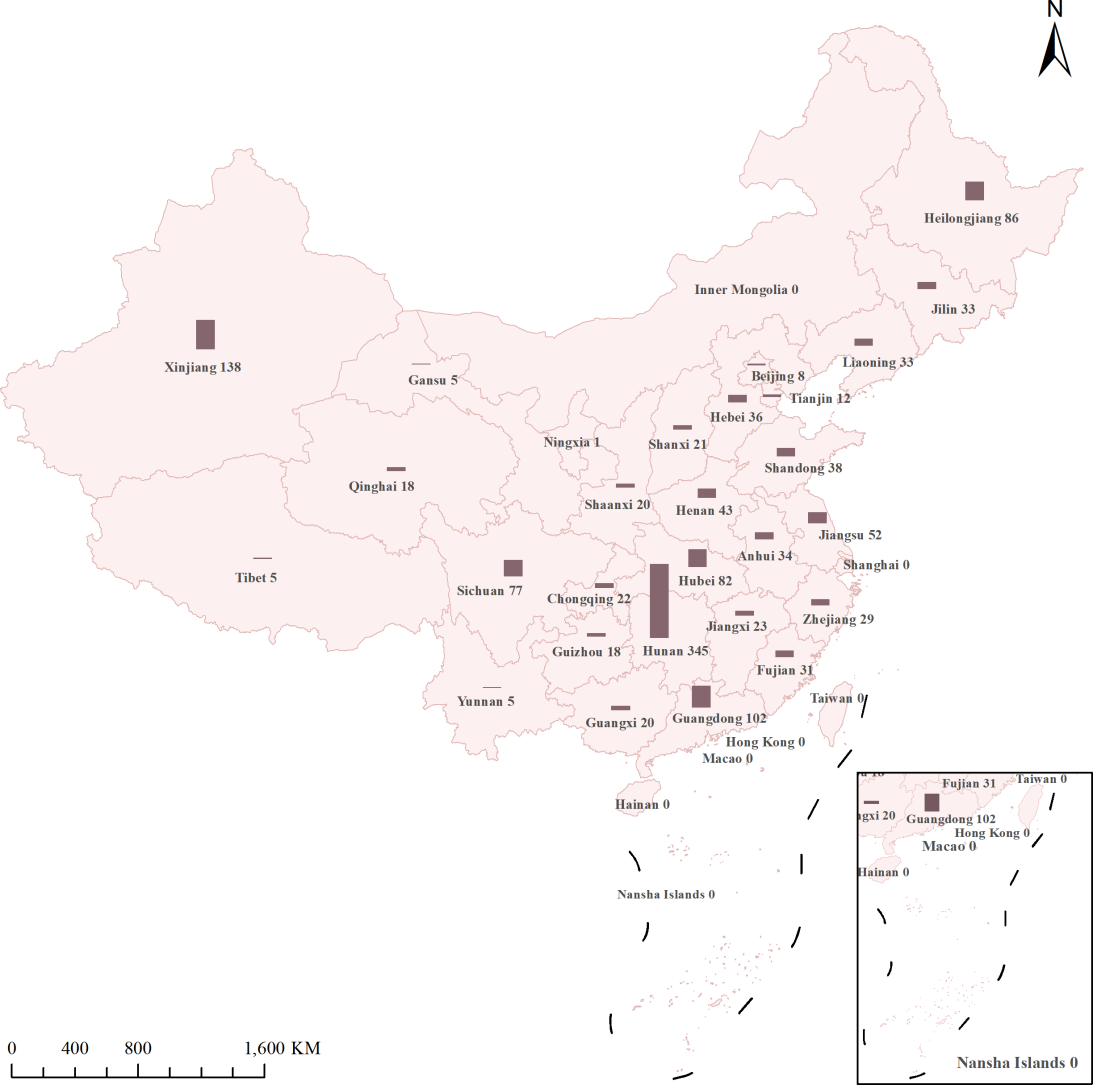
Sampled INH^r^-MTB isolates from various geographic regions in China.

A total of 1,337 INH^r^-MTB isolates identified using 1% proportion method on Löwenstein-Jensen (L-J) medium were collected from 22 provinces, three municipalities, and three autonomous regions through the Chinese Drug Resistance Surveillance Program (14) from 2013 to 2020 (Fig. 1).

### Broth microdilution DST

Phenotypic drug susceptibility testing (DST) was conducted using the broth microdilution method with commercial MYCOTB plates (Thermo Fisher Scientific, USA) to assess the minimal inhibitory concentrations (MICs) of ETO, INH, and rifampicin (RIF). Briefly, the bacterial clumps from L-J medium were disaggregated through ultrasonic treatment (30 s pulses at 40 kHz), followed by turbidity adjustment to 0.5 McFarland standard using a densitometer. For inoculum preparation, 100 μL of the homogenized suspension was diluted in 10 mL of Middlebrook 7H9 broth supplemented with 10% OADC enrichment. Using an automated liquid handling system (BioMek 4000), 100 μL aliquots of this standardized inoculum were precisely dispensed into each well of the MYCOTB microplate, which contained serial drug dilutions. After sealing with breathable membranes, the plates were incubated at 37°C in 5% CO₂ for 10–14 days. MIC values were determined visually as the lowest drug concentration showing complete growth inhibition, with results validated against the reference strain *M. tuberculosis* H37Rv (ATCC 27294). The clinical breakpoints were defined as: 0.12 μg/mL for INH, 0.5 μg/mL for RIF, and 4 μg/mL for ETO (15). Isolates demonstrating INH MICs ≥ 2 µg/mL were classified as high-level resistant (16). MDR isolates were defined as resistance to at least INH and RIF. Isolate information, including MICs and mutations, is shown in Table S5.

### WGS analysis

Genomic DNA was extracted from *Mycobacterium tuberculosis* isolates cultured on L-J medium using the cetyltrimethylammonium bromide (CTAB) method. DNA samples meeting quality thresholds were submitted to Annoroad Gene Technology (Beijing, China) for WGS using the Illumina HiSeq 2500 platform (150 bp paired-end reads). Initial quality assessment of raw sequencing data was performed using FastQC (v0.11.8), followed by adapter trimming and quality filtering with Trimmomatic (v0.38) under default parameters with a minimum Phred quality score threshold of 20. Potential human host contamination was removed by aligning reads to the GRCh38 reference genome and excluding matching sequences. The remaining high-quality reads were aligned to the *M. tuberculosis* H37Rv reference genome (GenBank ID: NC_000962.3). Variant calling was performed using SAMtools (v1.3.1) and GATK (v3.8.0) to identify single-nucleotide polymorphisms (SNPs) and insertion/deletion (indel) mutations. Finally, all detected variants were cross-referenced with the WHO mutation catalog to identify known and putative drug resistance-associated mutations.

### Sequencing of fragments of *ahpC*, *inhA*, and *katG* genes

Boiling method was used for crude DNA extraction from sputum for sequencing. The DNA was amplified with primers shown in Table S1 and subjected to sequencing for *ahpC*, *inhA*, and *katG* gene fragments. The sequencing results were compared with the H37Rv sequence. Significantly, the nomenclature in our study is different from the WHO catalog (17), and some of the *inhA* mutations that occurred in the *fabG1-inhA* operon have the alias with *fabG1* (18–20), so that we provide the reference table of the different nomenclature in Table S3.

## RESULTS

### Data description

**Fig 2:**
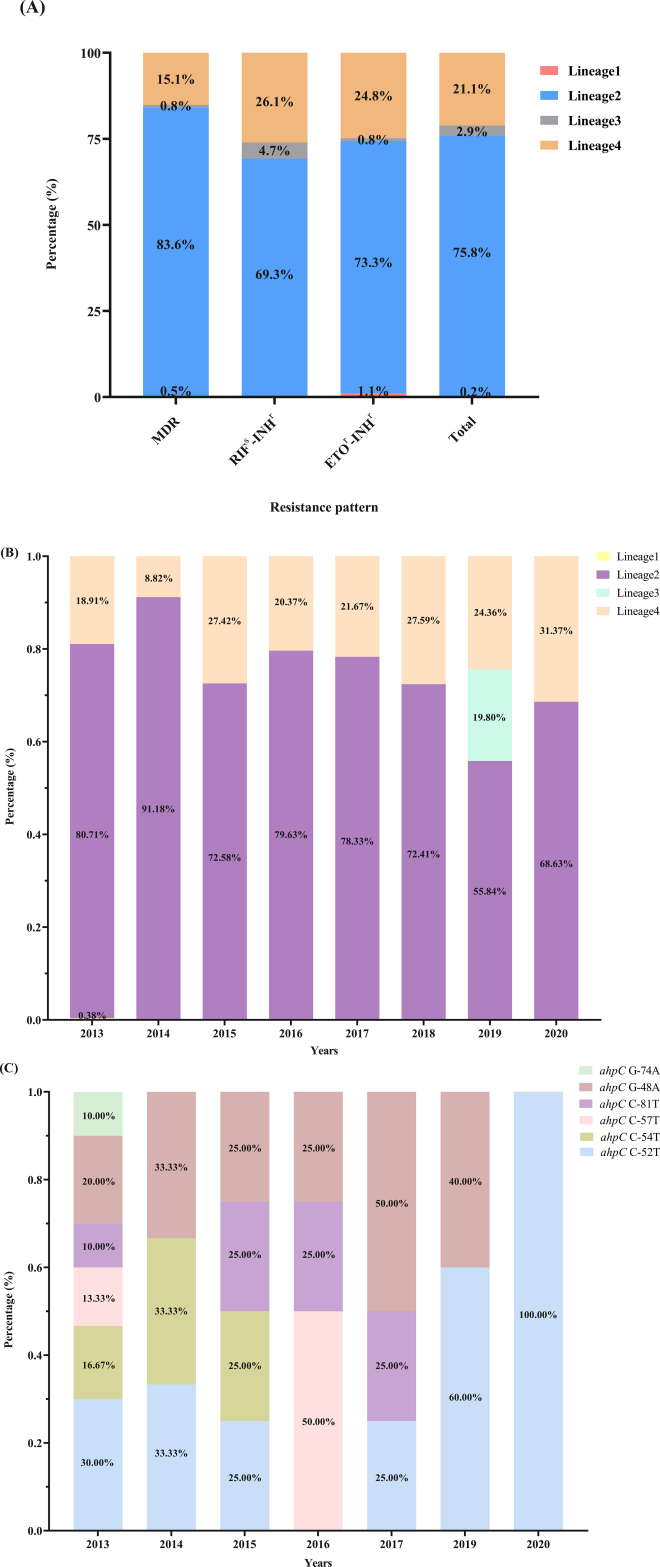
Spectrum description of 1,337 *Mycobacterium tuberculosis* isolates in drug susceptibility patterns and variant distribution in years. (**A**) Drug susceptibility patterns of 1,337 INH-resistant *Mycobacterium tuberculosis* isolates. (**B**) Ratio of different lineages every year in 2013–2020 (ratio = numbers of the specific lineage/the total numbers of strains in specific year). (**C**) Ratio of *ahpC* mutations distribution every year in 2013–2020. No *ahpC* variants existed in 2018 that have not been shown in the figure (ratio = numbers of the specific mutation type/the total numbers of *ahpC* variants in specific year).

Among all 1,337 INH^r^-MTB clinical isolates, the proportions of MDR (RIF^r^-INH^r^) and RIF^s^ (RIF^s^-INH^r^) were 45.5% (608/1,337) and 54.5% (729/1,337), respectively, with 19.9% (266/1,337) presenting resistant to ETO (ETO^r^-INH^r^) as well. The proportions of the four MTB lineages were identified with 75.8% lineage 2 (L2) (1,013/1,337), 21.1% lineage 4 (L4) (282/1,337), 2.9% lineage 3 (L3) (39/1,337), and 0.2% lineage 1 (L1) (3/1,337) (Fig. 2A) (Table S2). In 2013–2020, lineage 2 has been in the majority of strains in each year, followed by lineage 4. Lineage 1 was identified only in 2013 with the proportion of 0.38% (3/788), while lineage 3, which existed only in 2019, was identified with 19.80% (39/197) (Fig. 2B) (Table S2). In regard to different *ahpC* variants in 2013–2020, *ahpC* G-48A (2013–2017, 2019) and C-52T (2013–2015, 2017, 2019–2020) were both identified in 6 years, followed by *ahpC* C-81T (2013, 2015–2017) in 4 years and C-54T (2013–2015) in 3 years. *ahpC* C-57T (2013, 2016) was identified in 2 years, while G-74A only existed in 2013 (Fig. 2C) (Table S2). In 2013–2017, more than three types of *ahpC* variants consisted of the mutation spectrum in specific years, while there were only two and a single type of variants in 2019 and 2020, respectively, and no *ahpC* variants were identified in 2018 (Fig. 2C).

### Characteristics of isoniazid resistance-conferring mutations

Among the 1,337 strains analyzed, 1,058 (79.1%) carried mutations in the *ahpC*, *katG*, and *inhA* genes. Of these, the majority (71.4%, 755/1,058) exclusively harbored *katG* mutations, while 21.0% (222/1,058) and 5.3% (56/1,058) carried mutations solely in *inhA* and *ahpC*, respectively. The most prevalent mutation was *katG* Ser315Thr (65.5%, 693/1,058), followed by *inhA* C-777T (19.7%, 208/1,058) and *katG* Ser315Asn (5.4%, 57/1,058). Additionally, 2.4% (25/1,058) of strains exhibited double-site mutations, with the most common combination being *katG* Ser315Thr and *inhA* C-777T (1.1%, 12/1,058). Among the 266 INH^r^-ETO^r^ isolates, 208 (78.2%) carried mutations in *ahpC*, *katG*, or *inhA*. The predominant mutation in this subset was *inhA* C-777T (74.5%, 155/208), followed by *katG* Ser315Thr (13.9%, 29/208) and *inhA* G-154A (1.9%, 4/208) (Table 1).

**TABLE 1:** Genetic analysis of the INH^r^-MTB isolates^*a*^

Mutation type	Total (%)	MDR (%)	RIF^s^ (%)	ETO^r^ (%)
*ahpC* C-52T	19 (1.8)	14 (1.3)	5 (0.5)	0 (0)
*ahpC* G-48A	15 (1.4)	8 (0.8)	7 (0.7)	1 (0.5)
*ahpC* C-54T	7 (0.7)	4 (0.3)	3 (0.3)	0 (0)
*ahpC* C-57T	6 (0.6)	5 (0.5)	1 (0.1)	1 (0.5)
*ahpC* C-81T	6 (0.6)	3 (0.3)	3 (0.3)	0 (0)
*ahpC* G-74A	3 (0.3)	3 (0.3)	0 (0)	1 (0.5)
*katG* Ser315Thr	693 (65.5)	344 (32.5)	349 (33.0)	29 (13.9)
*katG* Ser315Asn	57 (5.4)	23 (2.1)	34 (3.2)	3 (1.4)
*katG* Ser315Arg	3 (0.3)	2 (0.2)	1 (0.1)	0 (0)
*katG* Ser315Ile	2 (0.2)	1 (0.1)	1 (0.1)	0 (0)
*inhA* C-777T	208 (19.7)	52 (4.9)	156 (14.8)	155 (74.5)
*inhA* G-154A	12 (1.1)	7 (0.7)	5 (0.5)	4 (1.9)
*inhA* T-770A	2 (0.2)	2 (0.2)	0 (0)	1 (0.5)
*ahpC* C-54T+*katG* Ser315Thr	1 (0.1)	0 (0)	1 (0.1)	0 (0)
*ahpC* G-48A+*katG* Ser315Thr	1 (0.1)	1 (0.1)	0 (0)	0 (0)
*ahpC* C-52T+*inhA* C-777T	1 (0.1)	0 (0)	1 (0.1)	1 (0.5)
*ahpC* G-74A+*inhA* C-777T	1 (0.1)	0 (0)	1 (0.1)	1 (0.5)
*ahpC* G-74A+*inhA* T-770C	1 (0.1)	0 (0)	1 (0.1)	0 (0)
*ahpC* C-54T+*ahpC* C-39T	1 (0.1)	1 (0.1)	0 (0)	0 (0)
*ahpC* C-81T+*ahpC* C-52T	1 (0.1)	1 (0.1)	0 (0)	0 (0)
*ahpC* C-81T+*ahpC* G-74A	1 (0.1)	1 (0.1)	0 (0)	0 (0)
*katG* Ser315Thr+*inhA* C-777T	12 (1.1)	10 (0.9)	2 (0.2)	9 (0.4)
*katG* Ser315Thr+*inhA* G-154A	2 (0.2)	2 (0.2)	0 (0)	1 (0.5)
*katG* Ser315Thr+*inhA* T-770C	2 (0.2)	1 (0.1)	1 (0.1)	0 (0)
*katG* Ser315Thr+*inhA* Ser94Ala	1 (0.1)	1 (0.1)	0 (0)	1 (0.5)
Total	1,058 (100)	486 (45.9)	572 (54.1)	208 (19.7)

^*a*^ Cross resistance: INH^r^-MTB strains containing non-INH resistance-related mutations.

### Correlation of *ahpC* mutations with INH MICs

Due to the small number of strains with combined mutations in *ahpC*, along with *katG*, *inhA* or double-site mutations were categorized as “others.” Accordingly, 83.3% (5/6) C-81T, 60.0% (9/15) G-48A, 57.1% (4/7) C-54T, 50.0% (3/6) C-57T, and 42.1% (8/19) C-52T mutations were associated with high-level resistance to INH (MIC ≥ 2 µg/mL), while all of the *ahpC* C-57T mutants presented as MDR, and none of the *ahpC* G-74A mutants showed high-level resistance to INH (Table 2).

**TABLE 2:** Analysis of the high-level INH-resistant MTB isolates^*a*^^,^^*b*^

Mutation type	High-level INH^r^-MTB isolates	MDR	INH^r^-RIF^s^
*ahpC* C-52T	42.1% (8/19)	31.6% (6/19)	10.5% (2/19)
*ahpC* G-48A	60.0% (9/15)	40.0% (6/15)	20.0% (3/15)
*ahpC* C-54T	57.1% (4/7)	28.6% (2/7)	28.6% (2/7)
*ahpC* C-57T	50.0% (3/6)	50.0% (3/6)	0
*ahpC* C-81T	83.3% (5/6)	50.0% (3/6)	33.3% (2/6)
others	50.0% (4/8)	25.0% (2/8)	25.0% (2/8)
*katG* Ser315Thr	77.1% (534/693)	37.2% (258/693)	39.8% (276/693)
*inhA* C-777T	6.3% (13/208)	4.8% (10/208)	1.4% (3/208)
*katG* Ser315Asn	56.1% (32/57)	19.3% (11/57)	36.8% (21/57)
*inhA* G-154A	8.3% (1/12)	8.3% (1/12)	0
*katG* Ser315Thr+*inhA* C-777T	91.7% (11/12)	75.0% (9/12)	16.7% (2/12)
*katG* Ser315Thr+*inhA* G-154A	100.0%	100.0%	0
*katG* Ser315Thr+*inhA* T-770C	50.0% (1/2)	0	50.0% (1/2)

^*a*^ High-level INH^r^-MTB isolates: MIC ≥ 2 µg/mL.

^*b*^ “others” refers to a small number of strains with combined mutations in *ahpC* along with *katG*, *inhA*, or double-site mutations.

### Correlation of *katG* and *inhA* mutations with INH MICs

**Fig 3:**
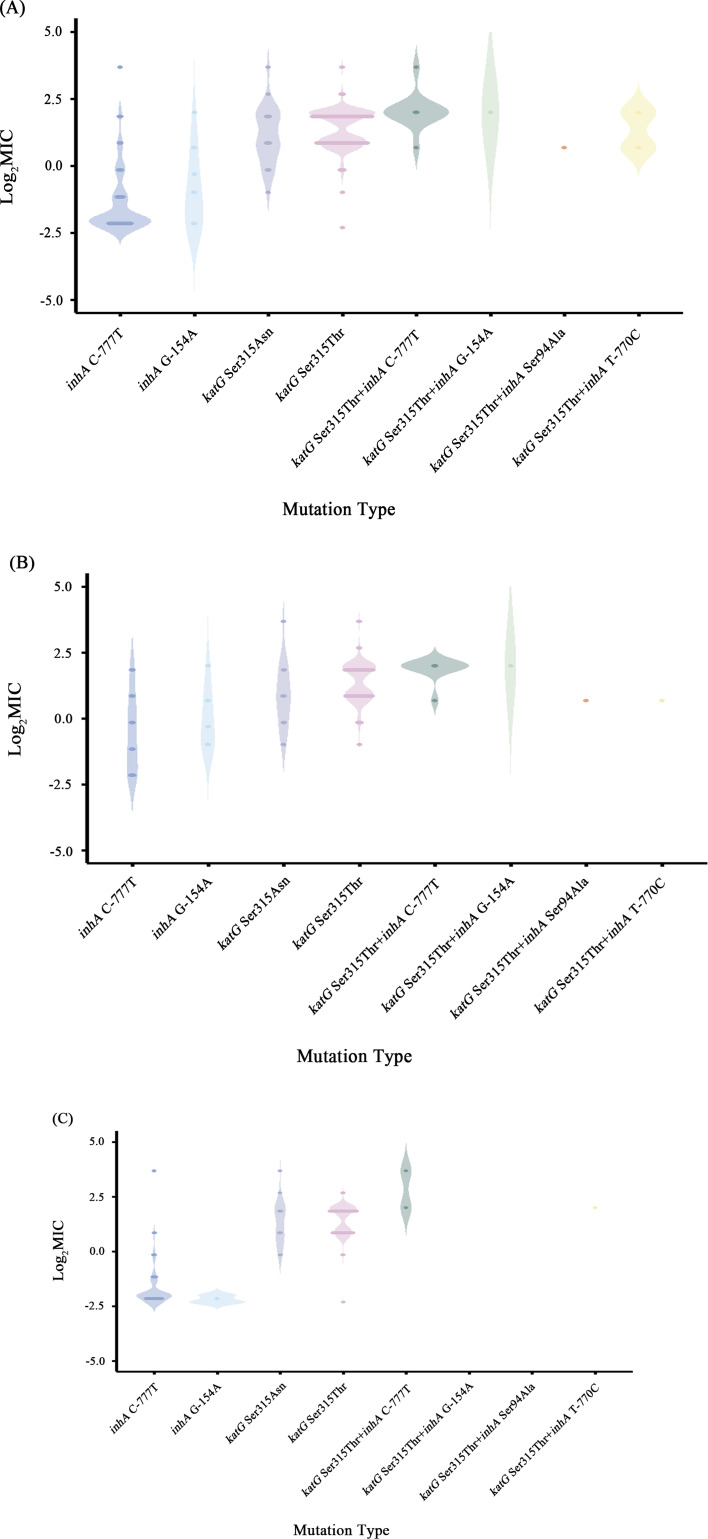
Distribution and log2MIC of different general INH-resistant mutations of the INH^r^-MTB isolates. The “log2MIC” refers to the logarithm of 2 for briefly comparing the MICs between isolates and the high-level INH-resistant threshold (MIC ≥ 2 μg/mL). (**A**) Distribution of the total of 1,337 INH^r^-MTB isolates. (**B**) Distribution of the MDR-MTB isolates. (**C**) Distribution of the RIFs-INH^r^-MTB isolates.

The log_2_MICs of INH^r^-related mutants in *katG* and *inhA* gene are shown in Fig. 3 and Table 2. With that, 91.7% (11/12) *katG* Ser315Thr+*inhA* C-777T, 77.1% (534/693) *katG* Ser315Thr, 56.1% (32/57) *katG* Ser315Asn, 50.0% (1/2) *katG* Ser315Thr+*inhA* T-770C, 8.3% (1/12) *inhA* G-154A, and 6.3% (13/208) *inhA* C-777T mutations were associated with high-level resistance to INH. All of the *katG* Ser315Thr+*inhA* G-154A and *inhA* G-154A mutants presented as high-level INH resistance, and all of the high-level INH^r^ strains in *katG* Ser315Thr+*inhA* T-770C mutants showed sensitive to RIF (INH^r^-RIF^s^), while none of the *katG* Ser315Thr+*inhA* Ser94Ala mutants were associated with high-level resistance to INH (Fig. 3; Table S3).

## DISCUSSION

The rise of isoniazid-resistant and multidrug-resistant tuberculosis threatens global TB control (1, 2, 5, 21). Key resistance mechanisms include *katG*-mediated catalase-peroxidase inactivation (16) and *inhA* promoter mutations disrupting INH-NAD adduct formation (18). Notably, compensatory *ahpC* promoter mutations in *katG*-deficient strains restore oxidative stress defenses via AhpC upregulation (10, 11). Here, we integrated drug susceptibility testing and WGS to analyze 1,337 INH^r^-MTB isolates, systematically characterizing INH resistance-associated genetic variants and their associations with isoniazid resistance levels.

Consistent with previous research (12, 22), our study found *katG* mutations (71.4%) to be predominant in INH^r^ isolates, with *katG* Ser315Thr (65.5%) as the primary resistance determinant, comparable to another Chinese report (63.0%) (23). Global epidemiological data reveal significant geographic variation in codon 315 mutation frequencies, ranging from 13 to over 90% (21, 24–27). The evolutionary predominance of *katG*^315^ mutations likely stems from their unique biochemical properties, which enable these variants to maintain sufficient catalase-peroxidase activity for oxidative protection and bacterial fitness while reducing INH activation capacity, thereby preserving virulence and transmissibility (28, 29). Notably, we observed the Ser315Thr substitution in MDR-TB (32.5%) isolates was comparable with that of INH mono-resistant isolates (33%), contrasting with prior study (41% vs 65%) (12), which may contribute to the differences in strain genetic background, treatment history, or compensatory evolution (6–11, 21–23).

Most KatG mutations or deletions significantly impair its catalase/peroxidase activity, consequently reducing bacterial virulence (6, 14–16, 18, 22, 23). Compensatory upregulation of AhpC (alkylhydroperoxidase) has been proposed to restore this functional deficit (7–11). While *ahpC-oxyR* mutations occur infrequently based on available data, our study identified single *ahpC* mutations in 5.3% of INH-resistant isolates, independently of the “sufficiently frequent” *katG*/*inhA* mutations listed in the WHO catalog (17). Only 0.1% of *ahpC* mutations co-occurred with established INH^r^ *katG*/*inhA* mutations, suggesting *ahpC*-mediated resistance may operate through KatG-independent pathways. Particularly intriguing was the C-57T variant exclusively found in MDR strains, which may contribute to an adaptive response to concurrent INH-RIF pressure, or a structural modification optimizing AhpC function under multidrug stress conditions (15, 23, 25).

While multiple genetic loci contribute to INH resistance, high-level resistance typically results from the accumulation of mutations in *katG*, *inhA*, and *ahpC* (12, 13). Our study revealed that only 6.3–8.3% of isolates with *inhA* mutations exhibited high-level resistance, consistent with previous reports associating single *inhA* mutations with low-level resistance (12). Notably, resistance levels increased substantially when *inhA* mutations co-occurred with *katG*^315^ mutations. Interestingly, we observed high-level resistance (42.1–83.3%) in isolates harboring single *ahpC-oxyR* mutations. This aligns with previous findings reporting high-level resistance (11.2%) with either single *ahpC-oxyR* mutations or combinations of *ahpC-oxyR* and non-315 *katG* mutations. These results suggest that these less frequent mutations may accumulate progressively during the evolution of INH resistance (12, 28, 29). As current molecular diagnostics primarily target high-frequency *katG*/*inhA* mutations, potentially overlooked rare *ahpC*-driven resistance (17, 24–26) inadvertently promotes the expansion of undetected resistant strains. In light of this, we have further emphasized that future case-control studies are necessary to definitively establish the specificity and positive predictive value of *ahpC* mutations as resistance markers. Collectively, our findings provide a strong rationale for future studies to evaluate the diagnostic utility of *ahpC* promoter mutations.

### Conclusion

Our findings demonstrate that the *ahpC* mutations are associated with high-level INH resistance in variants without concurrent *katG* or *inhA* mutations. This finding significantly advances our understanding of TB resistance profiling, enabling more comprehensive detection of INH^r^-MTB and optimizing therapeutic strategies.

## Data Availability

The raw whole-genome sequencing data reported in this article have been deposited in the Genome Sequence Archive (GSA: CRA017099) in the National Genomics Data Center.

## SUPPLEMENTAL MATERIAL

Supplemental tables spectrum.02496-25-s0001.docx Tables S1 to S4.

Table S5 spectrum.02496-25-s0002.xlsx Information for 1,337 INH^r^-MTB isolates.

ASM does not own the copyrights to Supplemental Material that may be linked to, or accessed through, an article. The authors have granted ASM a non-exclusive, world-wide license to publish the Supplemental Material files. Please contact the corresponding author directly for reuse.
