# Supplementary material for: Prevalence and resistance spectrum of ahpC mutations in isoniazid-resistant Mycobacterium tuberculosis isolates
Source: Microbiol Spectr. 2026 Mar 19;14(4):e02496-25. doi: 10.1128/spectrum.02496-25 (PMC13055390; doi:10.1128/spectrum.02496-25)
Supplement: Supplemental tables — Tables S1 to S4. [file spectrum.02496-25-s0001.docx]

**Table S1**. Primers used for sequencing of *ahpC, inhA*, and *katG* gene

| Primers | Sequences（5’-3’） |
| --- | --- |
| ahpC-Seq-F | CACCGAGACCGGCTTCCGA |
| ahpC-Seq-R | ACCCGCCACTTGCCTGGGT |
| inhA-Seq-F | CTGAGTCACACCGACAAACG |
| inhA-Seq-R | TCACATTCGACGCCAAACAG |
| katG-Seq-F | GGTCACACTTTCGGTAAGA |
| katG-Seq-R | GCCGTCCTTGGCGGTGTA |

**Table S2.** Spectrum description of 1337 *Mycobacterium tuberculosis* isolates in drug susceptibility patterns and variants distribution in years.

| 1. Drug susceptibility patterns of 1337 *Mycobacterium tuberculosis* isolates | | | | | | | | | | | | | |
| --- | --- | --- | --- | --- | --- | --- | --- | --- | --- | --- | --- | --- | --- |
| Resistance pattern | | Number of isolates (%) | | | Lineage1  No. (%) | | | Lineage2  No. (%) | | Lineage3  No. (%) | | Lineage4  No. (%) | |
| MDR  (RIF^r^-INH^r^) | | 608 (45.5) | | | 3 (0.5) | | | 508 (83.6) | | 5 (0.8) | | 92 (15.1) | |
| RIF^s^-INH^r^ | | 729 (54.5) | | | 0 (0.0) | | | 505 (69.3) | | 34 (4.7) | | 190 (26.1) | |
| ETO^r^ | | 266 (19.9) | | | 3 (1.1) | | | 195 (73.3) | | 2 (0.8) | | 66 (24.8) | |
| Total (INH^r^) | | 1337 (100.0) | | | 3 (0.2) | | | 1013 (75.8) | | 39 (2.9) | | 282 (21.1) | |
| *RIF: Rifampicin; INH: Isoniazid; ETO: Ethionamide.  Ratios in the line “Total” refers to the number of specific lineage/the total isolates; ratios of different lineages in each resistance pattern refers to the number of specific lineage/the total isolates in specific resistance pattern. | | | | | | | | | | | | | |
| 1. Ratio of different lineages every year in 2013-2020. | | | | | | | | | | | | | |
| Years | | | Number of isolates (%) | | | Lineage1  No. (%) | | Lineage2  No. (%) | | Lineage3  No. (%) | | Lineage4  No. (%) | |
| 2013 | | | 788 (58.9) | | | 3 (0.4) | | 636 (80.7) | | 0 | | 149 (18.9) | |
| 2014 | | | 34 (2.5) | | | 0 | | 31 (91.2) | | 0 | | 3 (8.8) | |
| 2015 | | | 124 (9.3) | | | 0 | | 90 (72.6) | | 0 | | 34 (27.4) | |
| 2016 | | | 54 (4.0) | | | 0 | | 43 (79.6) | | 0 | | 11 (20.4) | |
| 2017 | | | 60 (4.5) | | | 0 | | 47 (78.3) | | 0 | | 13 (21.7) | |
| 2018 | | | 29 (2.2) | | | 0 | | 21 (72.4) | | 0 | | 8 (27.6) | |
| 2019 | | | 197 (14.7) | | | 0 | | 110 (55.8) | | 39 (19.8) | | 48 (24.4) | |
| 2020 | | | 51 (3.8) | | | 0 | | 35 (68.6) | | 0 | | 16 (31.4) | |
| Total | | | 1337 (100.0) | | | 3 (0.2) | | 1013 (75.8) | | 39 (2.9) | | 282 (21.1) | |
| *Ratios in the line “Total” refers to the number of specific lineage/the total isolates; ratios of different lineages in each lineage refers to the number of specific lineage/the total isolates in specific year. | | | | | | | | | | | | | |
| 1. Ratio of *ahpC* mutations distribution every year in 2013-2020. | | | | | | | | | | | | | |
| Years | Number of isolates (%) | | | *ahpC* C-52T  No. (%) | | | *ahpC* C-54T  No. (%) | *ahpC* C-57T  No. (%) | *ahpC* C-81T  No. (%) | | *ahpC* G-48A  No. (%) | | *ahpC* G-74A  No. (%) |
| 2013 | 30 (53.6) | | | 9 (30.0) | | | 5 (16.7) | 4 (13.3) | 3 (10.0) | | 6 (20.0) | | 3 (10.0) |
| 2014 | 3 (5.4) | | | 1 (33.3) | | | 1 (33.3) | 0 | 0 | | 1 (33.3) | | 0 |
| 2015 | 4 (7.1) | | | 1 (25.0) | | | 1 (25.0) | 0 | 1 (25.0) | | 1 (25.0) | | 0 |
| 2016 | 4 (7.1) | | | 0 | | | 0 | 2 (50.0) | 1 (25.0) | | 1 (25.0) | | 0 |
| 2017 | 4 (7.1) | | | 1 (25.0) | | | 0 | 0 | 1 (25.0) | | 2 (50.0) | | 0 |
| 2018 | 0 | | | 0 | | | 0 | 0 | 0 | | 0 | | 0 |
| 2019 | 10 (17.9) | | | 6 (60.0) | | | 0 | 0 | 0 | | 4 (40.0) | | 0 |
| 2020 | 1 (1.8) | | | 1 (100.0) | | | 0 | 0 | 0 | | 0 | | 0 |
| Total | 56 (100.0) | | | 19 (33.9) | | | 7 (12.5) | 6 (10.7) | 6 (10.7) | | 15 (26.8) | | 3 (5.4) |
| *Ratios in the line “Total” refers to the number of the specific variants/the total numbers of *ahpC* variants in specific year; ratios of different variants in each variant refers to the number of specific variant/the total *ahpC* in specific year. | | | | | | | | | | | | | |

**Table S3.** Reference table of different nomenclature

| Our study | Catalogue of mutations from WHO | Alias^*^ |
| --- | --- | --- |
| *katG* Ser315Thr | *katG*_p.Ser315Thr | - |
| *katG* Ser315Asn | *katG*_p.Ser315Asn | - |
| *katG* Ser315Arg | *katG*_p.Ser315Arg | - |
| *katG* Ser315Ile | *katG*_p.Ser315Ile | - |
| *inhA* C-777T | *inhA*_c.-777C>T | *fabG1*_c.-15C>T |
| *inhA* G-154A | *inhA*_c.-154G>A | *fabG1*_p.Leu203Leu |
| *inhA* T-770A | *inhA*_c.-770T>A | *fabG1*_c.-8T>A |
| *inhA* T-770C | *inhA*_c.-770T>C | *fabG1*_c.-8T>C |
| *inhA* Ser94Ala | *inhA*_p.Ser94Ala | - |

*Some of the *inhA* mutations have occurred in the *fabG1*-*inhA* operon, and the “Alias” refers to the alias of these types with *fabG1*.

**Table S4.** Distribution and log_2_MIC of different general INH-resistant mutations of the INH^r^-MTB isolates.

| MIC  (μg/mL) | Log_2_MIC | *inhA* C-777T | *inhA* G-154A | *katG* Ser315Asn | *katG* Ser315Thr | *katG* Ser315Thr+  *inhA* C-777T | *katG* Ser315Thr+  *inhA* G-154A | *katG* Ser315Thr+  *inhA* Ser94Ala | *katG* Ser315Thr+  *inhA* T-770C |
| --- | --- | --- | --- | --- | --- | --- | --- | --- | --- |
| 0.20 | -2.32 | 40 | 3 | 0 | 1 | 0 | 0 | 0 | 0 |
| 0.25 | -2.00 | 90 | 2 | 0 | 0 | 0 | 0 | 0 | 0 |
| 0.40 | -1.32 | 20 | 0 | 0 | 0 | 0 | 0 | 0 | 0 |
| 0.50 | -1.00 | 15 | 3 | 2 | 1 | 0 | 0 | 0 | 0 |
| 0.80 | -0.32 | 9 | 1 | 5 | 10 | 0 | 0 | 0 | 0 |
| 1.00 | 0.00 | 13 | 0 | 6 | 15 | 0 | 0 | 0 | 0 |
| 1.60 | 0.68 | 8 | 2 | 12 | 132 | 1 | 0 | 1 | 1 |
| 2.00 | 1.00 | 5 | 0 | 8 | 167 | 0 | 0 | 0 | 0 |
| 3.20 | 1.68 | 1 | 0 | 5 | 92 | 0 | 0 | 0 | 0 |
| 4.00 | 2.00 | 6 | 1 | 16 | 259 | 10 | 2 | 0 | 1 |
| 6.40 | 2.68 | 0 | 0 | 1 | 12 | 0 | 0 | 0 | 0 |
| 12.80 | 3.68 | 1 | 0 | 2 | 4 | 1 | 0 | 0 | 0 |
| Total | | 208 | 12 | 57 | 693 | 12 | 2 | 1 | 2 |

*Numbers under each mutation type refer to the number of variants in the corresponding MIC.
